# Deficiency of myostatin protects skeletal muscle cells from ischemia reperfusion injury

**DOI:** 10.1038/s41598-021-92159-2

**Published:** 2021-06-15

**Authors:** Christoph Wallner, Marius Drysch, Mustafa Becerikli, Sonja Verena Schmidt, Stephan Hahn, Johannes Maximilian Wagner, Felix Reinkemeier, Mehran Dadras, Alexander Sogorski, Maxi von Glinski, Marcus Lehnhardt, Björn Behr

**Affiliations:** 1 Department of Plastic Surgery, BG University Hospital Bergmannsheil, Ruhr University Bochum, Bürkle-de-la-Camp Platz 1, 44789 Bochum, Germany; 2 Department of Molecular Gastrointestinal Oncology, Ruhr University Bochum, Universitätsstraße 150, 44780 Bochum, Germany

**Keywords:** Molecular medicine, Cell death, Cell signalling, Translational research

## Abstract

Ischemia reperfusion (IR) injury plays a pivotal role in many diseases and leads to collateral damage during surgical interventions. While most studies focus on alleviating its severity in the context of brain, liver, kidney, and cardiac tissue, research as regards to skeletal muscle has not been conducted to the same extent. In the past, myostatin (MSTN), primarily known for supressing muscle growth, has been implicated in inflammatory circuits, and research provided promising results for cardiac IR injury mitigation by inhibiting MSTN cell surface receptor ACVR2B. This generated the question if interrupting MSTN signaling could temper IR injury in skeletal muscle. Examining human specimens from free myocutaneous flap transfer demonstrated increased MSTN signaling and tissue damage in terms of apoptotic activity, cell death, tissue edema, and lipid peroxidation. In subsequent in vivo* Mstn*^*Ln/Ln*^ IR injury models, we identified potential mechanisms linking MSTN deficiency to protective effects, among others, inhibition of p38 MAPK signaling and SERCA2a modulation. Furthermore, transcriptional profiling revealed a putative involvement of NK cells. Collectively, this work establishes a protective role of MSTN deficiency in skeletal muscle IR injury.

## Introduction

Skeletal muscle IR injury is a multidisciplinary occurrence. While many studies have examined potential targets to alleviate tissue damage caused by IR, focus was often directed towards internal organs or cardiac muscle, respectively. Considering the high prevalence of skeletal muscle IR injury (e.g. surgery under tourniquet, free tissue transfer, acute arterial embolisms of extremities), scientific efforts do not seem commensurate with clinical significance. With cardiovascular diseases being among the leading causes of death in the western world^1^, it is obvious that most IR research focusses on the heart. However, occurrence of IR as side effect of elective surgery, may not be underestimated. Regarding microsurgical transfer of free tissue in humans, previous research has been reporting opposing results. While it was mentioned that usual time frames in free tissue transfer were too short to induce IR injury on a posttranslational level^2^, others demonstrated contrary evidence^3^. IR injury has been linked to free flap failure^4^, and, additionally, its importance has been illuminated in the context of successful vascularized composite allotransplantation^5^. In general, severe IR injury may even lead to remote organ damage potentially culminating in systemic inflammation and, at worst, death^6,7^.

Starting point of the pathophysiological cascade of IR injury^8–11^ is hypoxia that in turn interrupts the electron chain and hence adenosine triphosphate (ATP) production in mitochondria. The following anaerobic metabolism further decreases ATP levels as well as antioxidative agents and induces dysfunction of different ion channels/pumps on mitochondria, endoplasmic reticulum, and on the cell surface. Accumulation of H^+^, Na^+^, and Ca^2+^ leads to decreased pH, hyperosmolarity, and cell swelling which impairs enzyme activity and leads to clumping of nuclear chromatin. Reperfusion then increases radical oxygen species (ROS) generation, facilitated by lowered antioxidative agents in ischemic cells. Elevated levels of ROS induce endothelial stress, lipid peroxidation, cytokine release, DNA damage, complement activation, and cell membrane damage. If cell death occurs in the setting of IR injury it is generally a combination of concurrent processes; the most common ones are apoptosis, mitoptosis, necrosis, necroptosis, and autophagy.

Myostatin also known as growth differentiation factor 8 (GDF8) is encoded by the *MSTN* gene in humans and is a member of the TGF-β family. In animals, lack or inhibition of MSTN leads to increased muscle mass also referred to as “double muscling”^12^. A spontaneous *MSTN* mutation observed in a human individual had similar effects and resulted in gross muscle hypertrophy^13^. Besides phenotypic effects, MSTN interacts with vascular smooth muscle and inflammatory cells and has been linked to ROS modulation via interaction with NF-kB, TNF-α, and the NADPH oxidase system^14–17^. MSTN can further activate p38 mitogen-activated protein kinase (p38 MAPK)^15^, which is known to play a crucial role in IR injury^18^. In 2019, Magga et al. also demonstrated myocardial protection from IR injury by systemic blockade of MSTN receptor ACVR2B^19^. While these effects have been reported in the heart, pathophysiological considerations suggest similar effects in skeletal muscle.

This work reveals the significance of IR injury in routinely performed free myocutaneous flap surgery and investigates a potential role of MSTN deficiency in mitigating skeletal muscle cell damage arising from IR utilizing in vivo knockout studies.

## Results

### Impact of IR injury on human skeletal muscle

**Figure 1:**
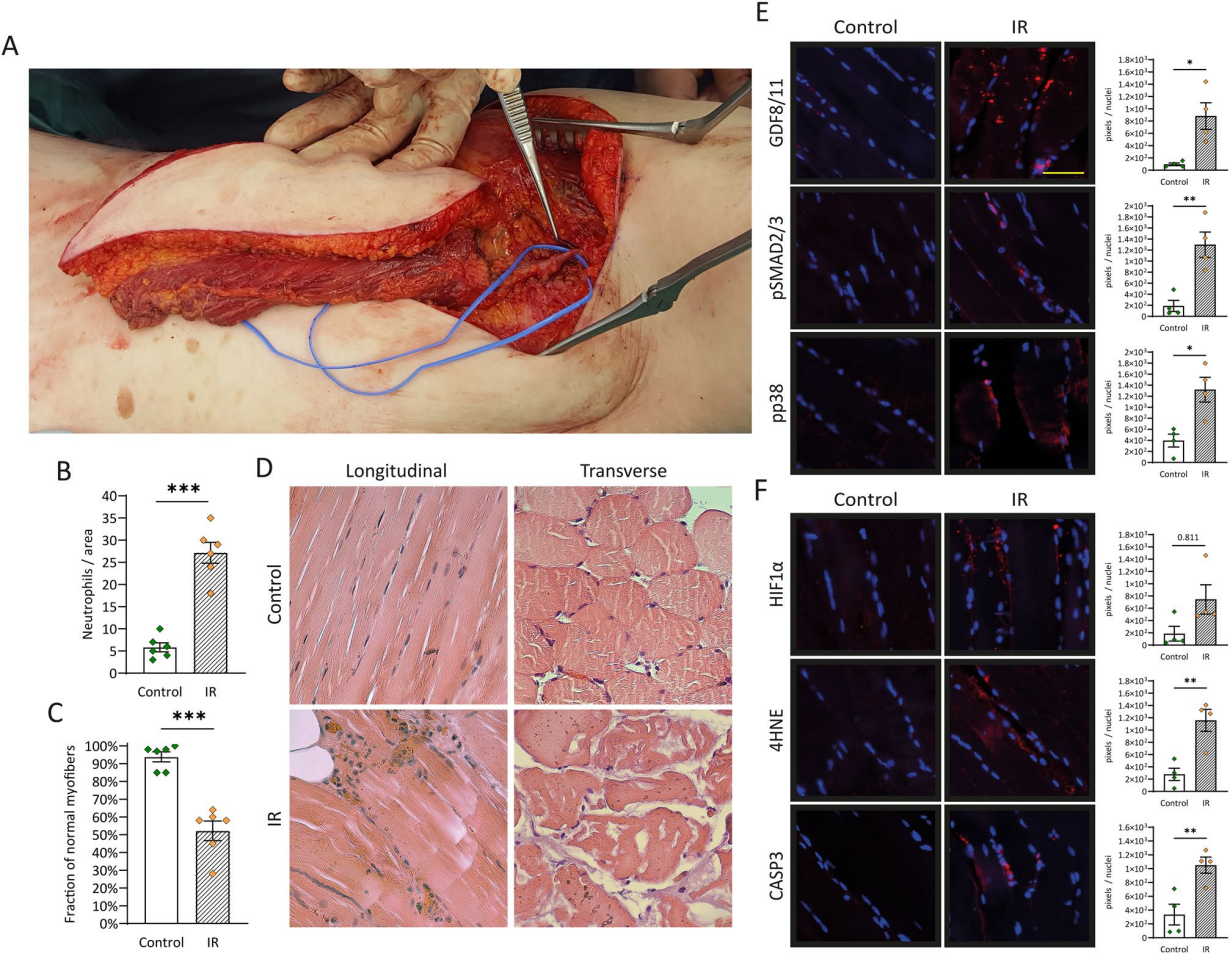
Impact of IR injury on human skeletal muscle cells. (**A**) Harvested free latissimus dorsi myocutaneous flap from which muscle specimens were obtained. (**B**) Infiltration of inflammatory cells into skeletal muscle and (**C**) magnitude of myofiber deterioration assessed in HE stained specimens. (**D**) representative images in longitudinal and cross-sectional views (**E**) Quantification of protein levels of MSTN (GDF8) and its downstream targets phospho-SMAD2/3 (pSMAD2/3) and phospho-p38 MAPK (p38 MAPK). Left panel shows representative images of immunofluorescence analysis. (**F**) Markers of hypoxia, lipid peroxidation, and apoptosis quantified using immunofluorescence analysis. Left panel shows representative images. Control: specimens harvested immediately before blood circulation detachment of the latissimus dorsi muscle, IR: specimens harvested shortly before finishing surgery (mean ischemia time: 64 ± 9 min, mean reperfusion time: 77 ± 25 min). Results are shown as means ± SEM. Scale bar 40 μm. p value: *< 0.05, **< 0.01, ***< 0.001 (unpaired two tailed t test).

Many studies have characterized IR injuries in different species leading to different outcomes depending on species, tissue, severity, and time of ischemia. To establish a baseline of IR injury in human skeletal muscle cells for this work, we sought to determine the extent of IR injury resulting from free autologous tissue transfer. Therefore, we utilized the free latissimus dorsi myocutaneous flap in a rare human experiment setting (Fig. 1A). Specimens were collected immediately before flap harvesting (control) and on the last occasion before final wound closure (IR), resulting in a mean ischemia time of 64 ± 9 min, and mean reperfusion time of 77 ± 25 min. Due to IR injury, inflammatory cell infiltration increased almost fivefold (Fig. 1B) and fraction of normal myofibers decreased by 42 percentage points (Fig. 1C). Inflammatory cell infiltration was validated using MPO staining (Fig. S1). Because previous studies demonstrated a connection between IR injury, MSTN, and its downstream targets^19^ we sought to investigate these proteins. In particular, MSTN, SMAD2/3, and p38 MAPK have been described to interact in a mutually dependent manner and upon cell stress and ischemia^20,21^. We found all three of them to be activated due to IR injury (Fig. 1E) which is consistent with previous findings^22^. To further validate an IR injury state in our samples and to grade hypoxia as well as oxidational and apoptotic processes, HIF-1α, 4-hydroxynonenal, and caspase 3 were measured (Fig. 1F). HIF-1α is a well characterized protein upregulated in hypoxic conditions and displayed an almost fourfold increase (p < 0.05). Caspase 3, marking the final pathway of apoptosis, displayed the same increase (fourfold, p < 0.01), and 4-HNE as marker lipid peroxidation due to oxidative stress^23^ rose threefold (p < 0.01). Taken together, these findings clearly demonstrated the extent of IR injury in routinely performed free latissimus dorsi myocutaneous flap transfer which is paralleled by an increase in MSTN and its downstream targets, noteworthy p38 MAPK.

### Transcriptome changes in skeletal muscle following IR injury

**Figure 2:**
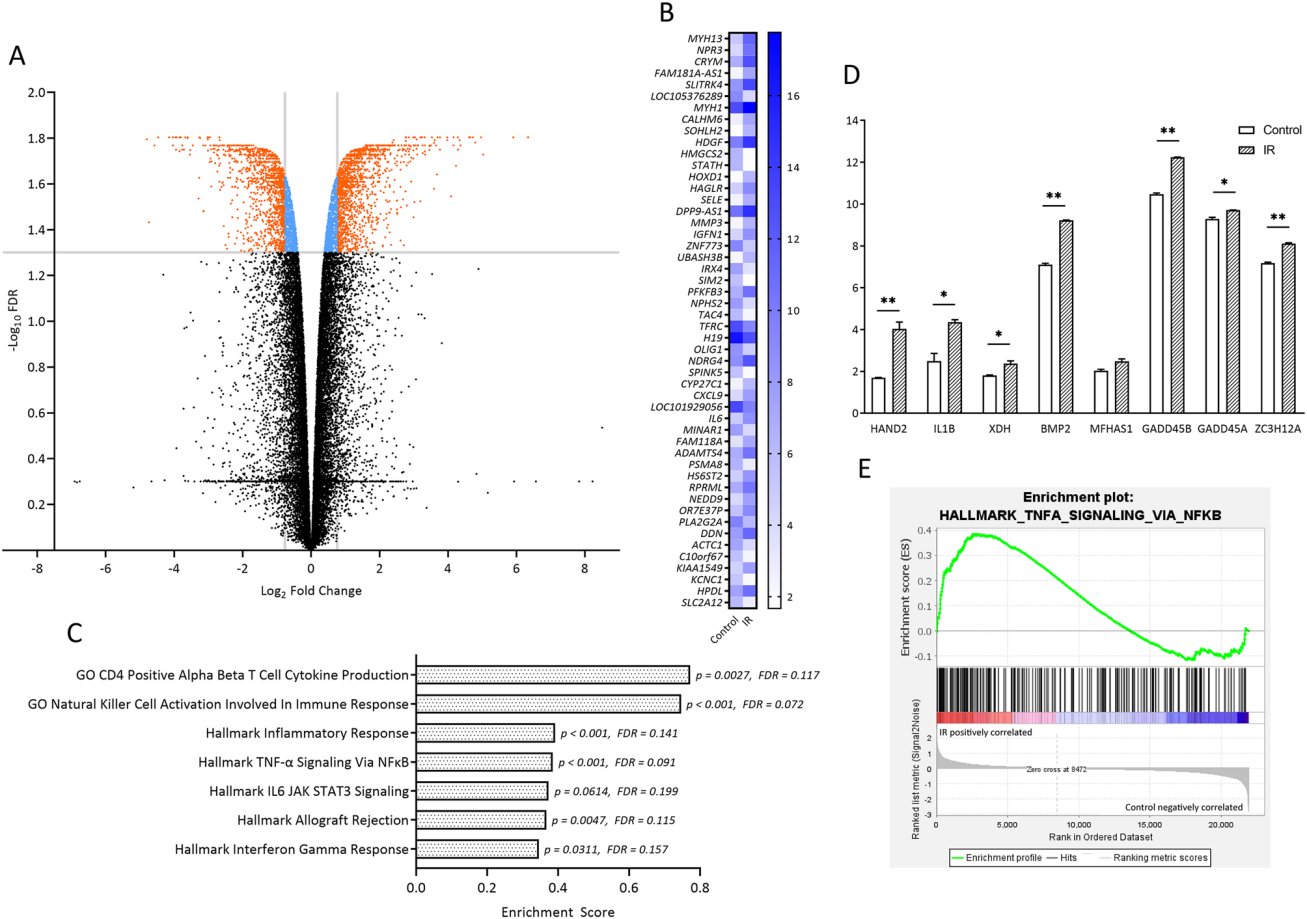
Transcriptional profiling of human IR injury. (**A**) Volcano plot representing differentially expressed genes (DEGs) characterized by expression values within the 50th–100th percentile, fold change ≥ 1.7 and corrected p value (Benjamin and Hochberg, FDR) < 0.05. (**B**) Heatmap with the 50 DEGs displaying the highest fold change. (**C**) Gene set enrichment analysis using MSigDB collections hallmarks gene sets and GO gene sets BP and MF. (**D**) Expression of enriched mRNAs in the positive regulation of p38 MAPK cascade gene set. FDR-values (Benjamini–Hochberg): *< 0.1; **< 0.05; (**E**) Enrichment plot of Hallmark TNF-α signaling via NF-κB (p < 0.001, FDR = 0.092). RNA of 4 biological samples per group was pooled considering concentration differences and analyzed in duplicates via RNA microarray (Agilent Technologies, California, USA).

Next, we examined the impact of IR injury on skeletal muscle on a transcriptional level. Microarray analysis revealed 2554 differentially expressed genes (DEGs, Fig. 2A) of which 1360 were up- and 1194 downregulated due to IR (Supplementary Dataset S1). Overexpression analysis with ConsensusPathDB mapped 1152 of the upregulated genes to 3102 1- and 2-next neighbours network neighbourhood-based entity sets (NESTs, filtered for p < 0.001, minimum overlap with input list = 5, Fig. S2A,B) and 45 annotated Kyoto Encyclopedia of Genes and Genomes (KEGG) pathways^24^ (filtered for p < 0.01, minimum overlap with input list = 5, Fig. S2C). Regarding the 148 1-next NESTs, most highly overrepresented set centers were HLA class II related (Fig. S2A), presumably reflecting the immune response to IR. Further overrepresented set centers included “NFkB.complex” and “p-S423, S425-SMAD3” of which the latter meets the observed immunofluorescence analysis upregulation (Fig. 1E). Overrepresented 2-next NESTs included 2954 set centers. Among these, several inflammatory markers (e.g. “IL6”, “NOD2”, “IFNG gene”) and p38 MAPK related proteins (“p38”, “mapk12”, “mapk13”) were found (Fig. S2B). Overrepresented KEGG pathways encompassed immunological pathways, i.e. “Graft-versus-host disease”, “Allograft rejection”, and interestingly, further autoimmune pathways (Fig. S2C), all in all indicating a strong immune response (Supplementary Dataset S2). The 50 DEGs with the highest fold change between groups (Fig. 2B) are in line with previous observations. MMP3, IL6, and CXCL9, for instance, have all been described in the realm of IR injury^6,25,26^. To further investigate the transcriptome, we performed Gene Set Enrichment Analysis (GSEA) including hallmark and Gene Ontology (GO) Biological Process (BP) and Molecular Function (MF) gene sets from MSigDB (Fig. 2C,E). Gene enrichment and level of significance were highest in gene sets “GO Natural Killer Cell Activation Involved In Immune Response” (ES: 0.75, p < 0.001, FDR = 0.072) and “Hallmark TNF-α Signaling Via NFkB” (ES: 0.38, p < 0.001, FDR = 0.091, Fig. 2E). Overall, enriched gene sets with an FDR < 0.1 aligned well with the analysis of DEGs and previous studies^27,28^, and convey the impression of a severe immune response, mediated via NK cells, TNF-α, NFkB, IL6, JAK1/2, STAT3 (Fig. 2C), and potentially p38 MAPK (Fig. 2D) in response to IR injury. Expression values from selected genes of the GO gene set “positive regulation of p38 MAPK cascade” (*IL1B, XDH, BMP2, GADD45B, GADD45A,* and *ZC3H12A*) are shown in Fig. 2D. Taken together, data obtained from analyzing human skeletal muscle (Figs. 1, 2) elucidated up-regulation of MSTN (GDF8), SMAD2/3, and p38 MAPK activity (and corresponding transcription), a severe immune response, and cell stress upon IR. Implications of these observations with particular focus on the interplay between MSTN, p38 MAPK, NFκB, and IL6 will be elaborated in “Discussion” section.

### MSTN knockout mitigates IR injury in murine skeletal muscle

**Figure 3:**
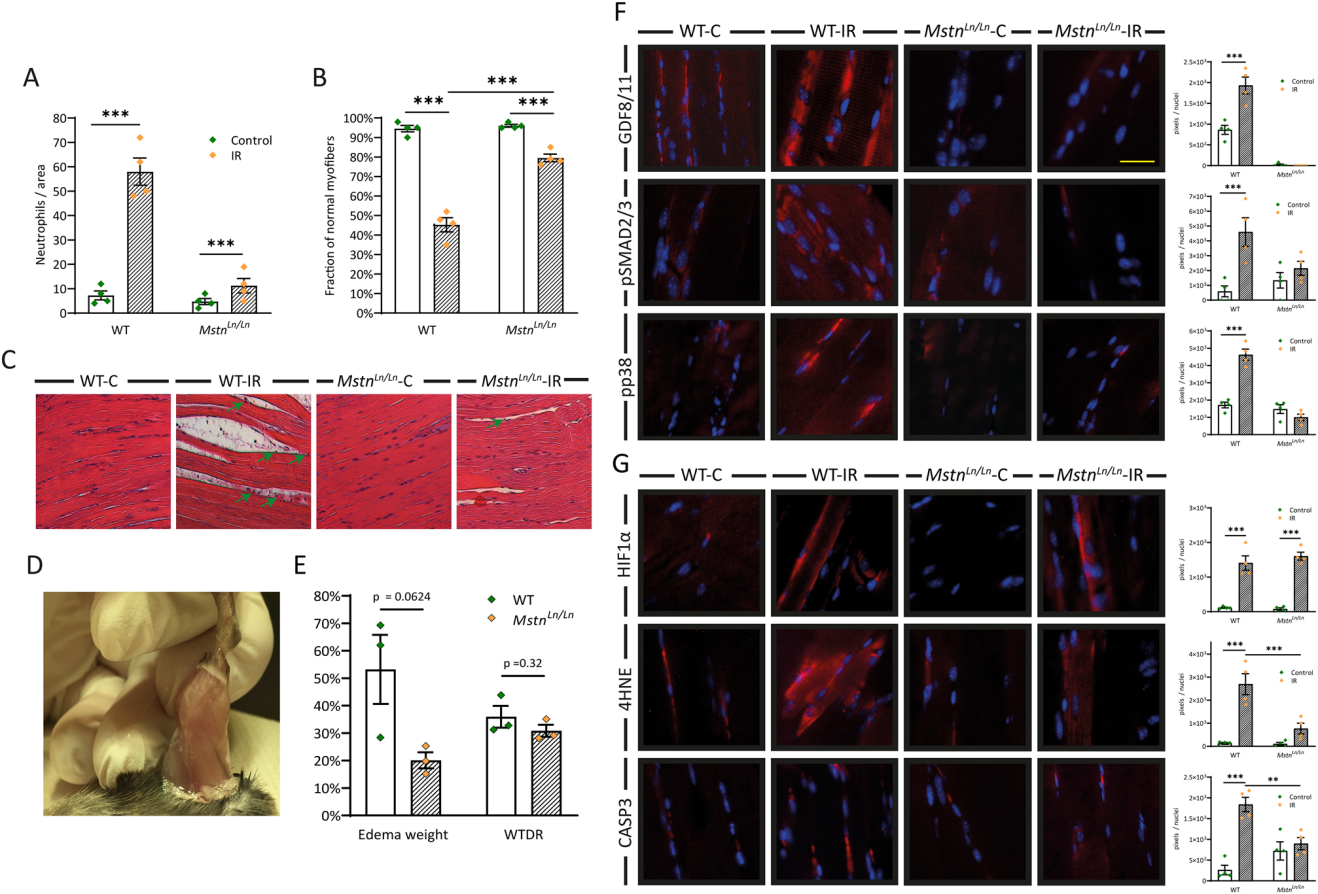
Mitigation of IR injury in MSTN deficient mice. (**A**) Tissue infiltration of inflammatory cells and (**B**) magnitude of myofiber deterioration assessed in HE stained skeletal muscle specimens of C57BL/6J and C57BL/6J-*Mstn*^*Ln/Ln*^ mice. (**C**) HE stained skeletal muscle demonstrating myofiber deterioration, neutrophil immigration (green arrows), and edema formation. (**D**) Macroscopically visible edema formation due to IR injury. (**E**) Changes in the wet-to-dry ratio and edema weight compared between C57BL/6J and C57BL/6J-*Mstn*^*Ln/Ln*^ mice (n = 3). For calculation of WTDR and edema weight see methods. (**F**) Immunofluorescence analysis of MSTN/GDF11-pSMAD2/3-pp38 MAPK pathway and representative images of immunofluorescence analysis. (**G**) Markers of hypoxia, lipid peroxidation, and apoptosis. Left panel illustrates representative images. WT = C57BL/6J mice, *Mstn*^*Ln/Ln*^ = C57BL/6J-*Mstn*^*Ln/Ln*^ mice. Results are shown as means ± SEM. Scale bar 40 μm. p value: *< 0.5, **< 0.1, ***< 0.001; two-tailed unpaired t test for pairwise analysis. ANOVA followed by multiple test comparison via Tukey’s post-hoc test (homoscedasticity) or Brown–Forsythe and Welch ANOVA followed by Dunnett T3 post-hoc test (heteroscedasticity) for multi-group analysis. RNA of four biological samples per group was pooled considering concentration differences and analyzed in duplicates via RNA microarray (Agilent Technologies, California, USA).

To further investigate a potential role of MSTN in IR injury, we used an in vivo knockout model. Heterozygote C57BL/6J-*Mstn*^*Ln/J*^ mice were obtained from Jackson Laboratory (Maine, USA, Stock No 009345) and bred to homozygote *Mstn*^*Ln/Ln*^ genotypes. Ischemia was induced as stated before^29^. Briefly, hindlimbs of both wildtype and *Mstn*^*Ln/Ln*^ were exposed to ischemia for 4 h using a low-pressure tourniquet. After reperfusion for 2 h, mice were euthanized, and the tissue was harvested. Human skeletal muscle had demonstrated neutrophil infiltration and myofiber deterioration in specimens due to IR injury (Fig. 1A,B). We thus started by examining the same aspects in mice. Number of neutrophils per area increased eightfold (p < 0.001, Fig. 3A,C) in WT mice; in *Mstn*^*Ln/Ln*^ mice on the other hand, neutrophil infiltration was not significant. Results were validated using MPO staining (Fig S4). Tissue deterioration was likewise reduced in *Mstn*^*Ln/Ln*^ mice. While WT mice exhibited signs of cell damage in almost 50% of previously healthy myofibers (p < 0.001), fraction of normal myofibers in *Mstn*^*Ln/Ln*^ mice only decreased by 16.5 percent points (p < 0.05, Fig. 3B,C). Regarding immunofluorescence analysis, MSTN deficiency further seemed to exert a protective role. Protein levels of MSTN (GDF8) (twofold), pSMAD2/3 (threefold), and pp38 MAPK (eightfold) were significantly increased in WT mice due to IR while the *Mstn*^*Ln/Ln*^ knockout blunted these effects (Fig. 3F,G). HIF-1α was substantially elevated in both groups, most certainly in response to hypoxic conditions. In WT mice IR led to increased lipid peroxidation (22-fold increase of 4HNE) and in turn to induction of apoptosis (sevenfold increase of caspase 3), grossly mimicking the events observed in human skeletal muscle. *Mstn*^*Ln/Ln*^ mice, however, displayed statistically insignificant enhanced lipid peroxidation (sevenfold increase of 4HNE), but this did not trigger apoptosis in terms of caspase 3 activity (Fig. 3F,G). To further elucidate the possibility of reduced apoptotic activity, we performed an apoptosis array (Fig. S3) which supported these observations. Decreased activity of caspase 3 upstream targets BAD (− 15%, p < 0.05) and BAX (− 27%, p < 0.01) in *Mstn*^*Ln/Ln*^-IR mice in comparison to WT-IR mice was accompanied by lowered levels of cleaved caspase 3 itself (− 22%, p < 0.01) (Fig. S3A). In addition, BCL-2 as prominent member of the antiapoptotic protein family was upregulated in response to IR injury in *Mstn*^*Ln/Ln*^ (+ 19%, p < 0.05) but not in WT mice (Fig. S3B). Besides apoptosis, another key element of IR injury is the inflammatory response. Here, diapedesis of neutrophils and upregulation of pro-inflammatory cytokines is regularly accompanied by interstitial fluid leakage^30^ which can be observed both microscopically (Fig. 3C) and macroscopically (Fig. 3D). Thus, in animal models of IR injury, it is a standard to evaluate edema formation. This was performed as previously described^29^. In brief, the hindlimb was cut off in toto, weighed, dried for 48 h, and weighed again. For exact calculation we kindly like to refer to “Materials and methods” section and supplementary dataset S3. Regarding the wet-to-dry-ratio, no statistically significant changes could be detected (Fig. 3E, right bars). Considering differences in weight between WT and muscular *Mstn*^*Ln/Ln*^ mice, the difference in edema increase between both groups is near-significant (p = 0.0624). Relative edema weight increased by 53% (± 12%) in WT mice and by only 20% (± 3%) in *Mstn*^*Ln/Ln*^ mice (Fig. 3E, left bars). Evaluating these results, one should also bear in mind the higher muscle mass and increased weight of *Mstn*^*Ln/Ln*^ mice which would let assume their proneness to IR injury and its sequelae, including edema formation. Hence, yet the non-distinctness in wet-to-dry-ratio may indicate diminished susceptibility to IR injury in *Mstn*^*Ln/Ln*^ mice.

### Transcriptome changes due to IR injury in WT and ***Mstn***^***Ln/Ln***^ mice

**Figure 4:**
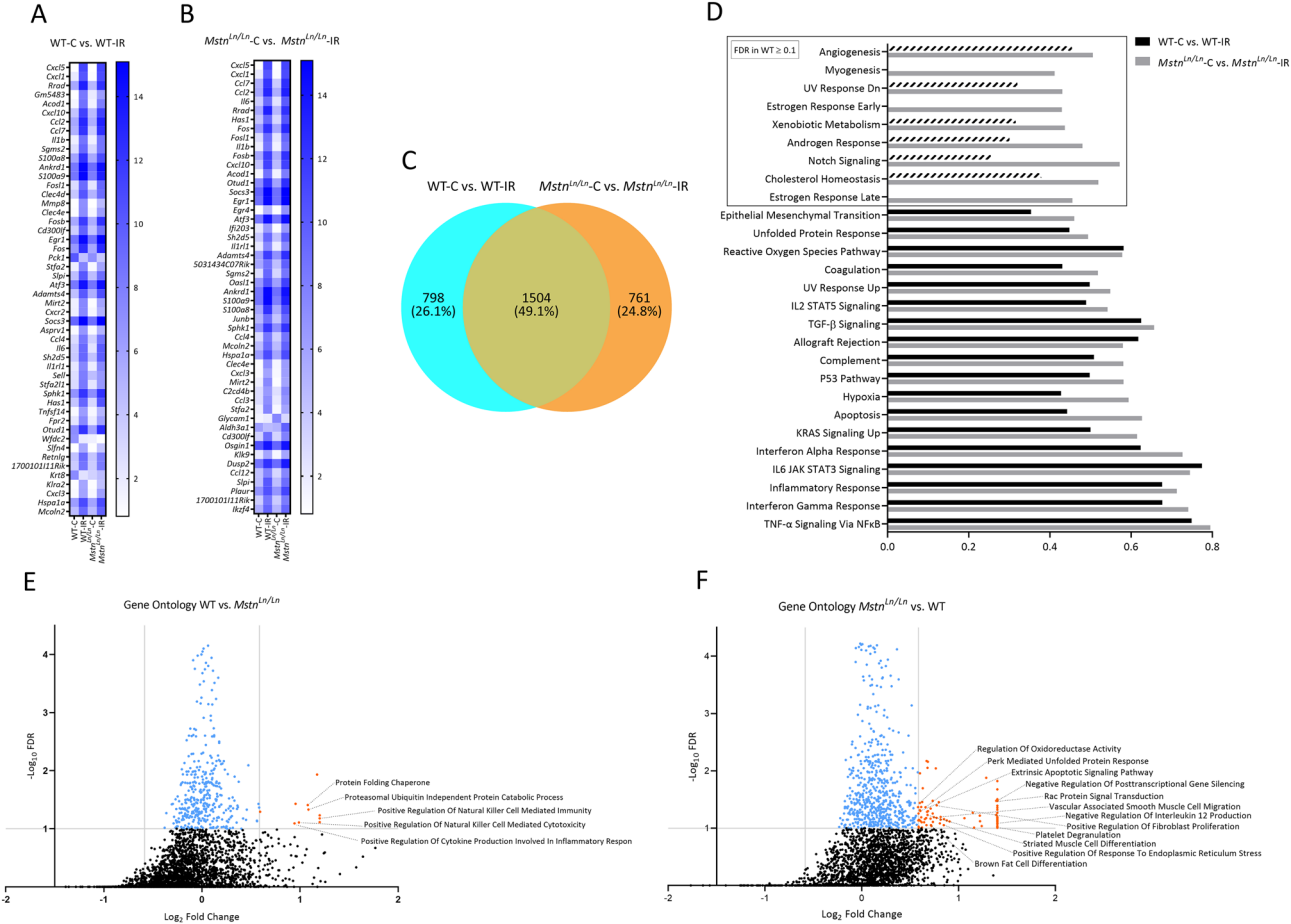
Transcriptional profiling of IR injury alleviation in WT *Mstn*^*Ln/Ln*^ mice. Heatmap depicting expression values of 50 DEGs (expression values within the 50th–100th percentile, fold change ≥ 1.7, FDR (Benjamini–Hochberg) < 0.05) sorted by descending fold change due to IR injury in (**A**) WT or (**B**) *Mstn*^*Ln/Ln*^ mice. (**C**) Venn diagram illustrating the overlap between genes that are differentially expressed due to IR injury in WT or *Mstn*^*Ln/Ln*^ mice, respectively. (**D**) GSEA with the hallmark gene set comparing enriched gene sets after IR injury in WT (WT-C vs. WT-IR) and *Mstn*^*Ln/Ln*^ (*Mstn*^*Ln/Ln*^-C vs. *Mstn*^*Ln/Ln*^-IR) mice. Enrichment scores inside the box are non-significant in WT mice at an FDR-threshold of 0.1. (**E**) GSEA of GO terms BP and MF comparing WT and *Mstn*^*Ln/Ln*^ mice before and after IR injury. Blue dots mark enrichment scores that are below the FDR-threshold of 0.1. Orange dots mark values that also meet the fold change cut-off of 1.5 when comparing the enrichment score of *Mstn*^*Ln/Ln*^-C vs. *Mstn*^*Ln/Ln*^-IR mice with the enrichment score of WT-C vs. WT-IR mice for a given gene set.

Since *Mstn*^*Ln/Ln*^ mice demonstrated increased tolerance towards IR injury, we sought to explore transcriptional changes using an RNA microarray (Agilent Technologies, California, USA). Upon analysis we identified 2303 DEGs in WT and 2261 DEGs in *Mstn*^*Ln/Ln*^ mice due to IR injury (Supplementary Dataset S4). Regarding the 50 DEGs with the highest fold change (Fig. 4A,B), results in both groups tied well with our transcriptome data of human IR injury (Fig. 2). DEGs can be mostly mapped to immunologic (e.g. *Cxcl5, Cxcl1, Cxcl10, Ccl7, Ccl2, Il6, Il1b*) and/or IR related activity (e.g. *Fos, Fosb, Fosl, Egr1, Mmp8, Acod1*)^31–34^. Considering the entire DEGs (Fig. 4C; Supplementary Dataset S4), both groups seemed to facilitate compensatory mechanisms in response to IR. Substantially elevated activation transcription factor 3 (WT: 54-fold change, FDR = 0.001; *Mstn*^*Ln/Ln*^: 53-fold change, FDR = 0.001), for instance, has been reported to convey protective effects against IR in different tissues^35^. Another example is *Dusp1* (WT: 1.9-fold change, FDR = 0.004; *Mstn*^*Ln/Ln*^: 3.9-fold change, FDR = 0.003). DUSP1 dephosphorylates members of the MAPK family and has been shown to ameliorate cardiac IR injury^36^. Stronger upregulation in *Mstn*^*Ln/Ln*^ mice also provided a potential explanation for decreased p38 MAPK (Fig. 3F) activity upon IR. To gain a better understanding of the DEGs, we examined the overlap between both groups (Fig. 4C; Supplementary Dataset S5). 798 genes were differentially expressed only in WT mice and 761 in *Mstn*^*Ln/Ln*^ mice. Shared DEGs added up to a total of 1504. We then mapped those that were upregulated in only one group to pathways using the ConsensusPathDB (filtered for p < 0.01, minimum overlap with input list = 5, Supplementary Dataset S6, S7). For WT mice, this once again verified the observations made during analysis of human IR, namely overrepresentation of pathways “NF-kappa B signaling pathways” and “Natural killer cell mediated cytotoxicity” (p < 0.001, Supplementary Dataset S6). Additionally, overrepresentation of the majority of the other 11 pathways (e.g., “Signaling by Interleukins”, “Cytokine Signaling in Immune system”, and “TYROBP Causal Network”) was consistent with pre-existing studies. For *Mstn*^*Ln/Ln*^ mice on the other hand, results differed substantially (Supplementary Dataset S7). Even though some of the 27 overrepresented pathways were related to enhanced immunologic activity, the vast majority was not. Instead, several pathways were related to MAPK signaling which might appear counterintuitive considering that our previous experiments demonstrated decreased expression of p38 MAPK. The MAPK family however incorporates several other MAP kinases as well as up- and downstream targets. For instance, further inspection of these gene sets revealed increased *Dusp4* and *Dusp16* expression. DUSP4 for its part has been linked to p38 MAPK regulation and is usually degraded under oxidative stress^37^. Furthermore, the set included *Rap1b*. While RAP1 has been linked to aggravated IR injury in the heart, the B-subunit has been shown to be imperative for angiogenesis^38^. Last pathway to emphasize is “VEGFA-VEGFR2 Signaling Pathway” which aligned well with the results of the GSEA (Fig. 4D–F). To conclude the analysis of DEGs, we filtered for genes that were differentially expressed in WT and *Mstn*^*Ln/Ln*^ mice but regulated differently, yielding 78 genes of interest (Fig. S5). Pathway analysis for DEGs up-regulated in WT and down-regulated in KO did not identify any overrepresentation. Vice versa, we determined 7 pathways containing DEGs down-regulated in WT mice but up-regulated in *Mstn*^*Ln/Ln*^ mice, mainly associated to muscle contraction (Supplementary Dataset S8). Five out of these incorporated ATPase Sarcoplasmic/Endoplasmic Reticulum Ca^2+^ Transporting 2 (*Atp2a2*). In WT mice, *Atp2a2* decreased 1.5-fold (FDR = 0.0018) upon IR injury; in *Mstn*^*Ln/Ln*^ on the contrary, it increased 2.2-fold (FDR = 0.0016). *Atp2a2* codes for SERCA2a, the most important SERCA genotype. It is mainly expressed in the heart and slow-twitch skeletal muscle. Upon activation, it can increase its activity to enhance Ca^2+^ uptake and thus reduce reperfusion-induced calcium overload. Moreover, studies have demonstrated proteolysis of SERCA2a, among others, via MMP-2, as part of cardiac IR injury^39^. Taken together, the increased expression of *Atp2a2* provides another potential mechanism that protects *Mstn*^*Ln/Ln*^ mice from IR injury. Upon deeper analysis, we identified further differently regulated mRNAs (*Krt8, Krt18, Mymx, Myh7b, Npr3*) between WR and *Mstn*^*Ln/Ln*^ mice that have been previously associated with IR injury and shall be examined in “Discussion” section. For more thorough transcriptome examination, we performed GSEA using Hallmark (Fig. 4D) and GO BP and MF (Fig. 4E,F) gene sets. Many enriched hallmark gene sets (Fig. 4D) were revealed before in human skeletal muscle upon IR, i.e. “TNF-α Signaling Via NFκB”, “Inflammatory response”, and “Allograft Rejection” (Fig. 2C). Strikingly, enriched hallmark gene sets in *Mstn*^*Ln/Ln*^ mice included “Myogenesis” (ES: 0.4, FDR = 0.088) without any enrichment in WT mice. Closer inspection of the data also indicated enrichment of gene sets “Angiogenesis” (ES: 0.5, FDR = 0.09), and “Notch Signaling” (ES: 0.57, FDR = 0.039) in *Mstn*^*Ln/Ln*^ mice but not in WT mice (ES: 0.45, FDR = 0.28 and ES: 0.25, FDR = 1, respectively). For GSEA, we filtered for gene sets displaying at least 1.5-fold change when comparing enrichment scores between WT-C vs. WT-IR and *Mstn*^*Ln/Ln*^-C vs. *Mstn*^*Ln/Ln*^-IR. FDR threshold was set to 0.1, resulting in 10 gene sets in WT and 76 gene sets in *Mstn*^*Ln/Ln*^ mice (Supplementary Dataset S9, S10). Interestingly, 3 gene sets enriched in WT mice were related to NK cell activation, one of the main enriched gene sets in human skeletal muscle (Fig. 2C). In line with this, “Negative Regulation of Interleukin 12 Production” (ES: 0.74, FDR = 0.078) was enriched in *Mstn*^*Ln/Ln*^ mice. Further enriched gene sets in *Mstn*^*Ln/Ln*^ mice were often related to cell differentiation and growth, all in all creating the impression of enhanced tissue proliferation in response to IR. Altogether, observed transcriptome changes supported the results presented in the previous section (Fig. 3) and identified several potential mechanisms of mitigated IR injury arising from *Mstn*^*Ln/Ln*^ deficiency.

## Discussion

This work emphasizes the incidence of skeletal muscle IR injury in routinely performed surgery and demonstrates protective effects of MSTN deficiency. Despite short time frames (mean ischemia time: 64 ± 9 min, mean reperfusion time: 77 ± 25 min), we found unequivocal signs of IR injury when examining skeletal muscle from free flap surgery. Observed increases in markers of hypoxia, lipid peroxidation, and apoptosis thereby corroborate previous findings in either skeletal muscle or other tissues^40–42^. Interplay between MSTN and p38 MAPK as well as their involvement in IR injury have likewise been stated before^20,21^. Elevated p38 MAPK has been described in cardiac ischemic preconditioning^43^ and p38 MAPK inhibition has been shown to alleviate myocardial IR^44^. Moreover, similar observations have been made in the context of other organs^45,46^. This work, however, is the first one to demonstrate a positive correlation between elevated levels of MSTN, p38 MAPK, and the presence of IR injury in human skeletal muscle. Transcriptional assessment revealing upregulation of genes included in the GO gene set “positive regulation of p38 MAPK cascade” further underlined the observed contribution of p38 MAPK. But p38 MAPK is not the only mediator linking MSTN to IR injury. NFκB is a transcription factor with basically ubiquitous occurrence that can be activated by several stimuli. It is also well-established that it can be stimulated via TNF-α and other chemokines such as IL-6. In 2011, Sriram et al.^15^ revealed that Myostatin itself can activate NFκB which in turn enhances transcription of Myostatin and TNF-α, further increasing NFκB activity in the sense of a positive feedback loop. Additionally, TNF-α as well as IL-6 can also directly activate transcription of MSTN^14^. In line with our hypothesis of a protective role of MSTN deficiency, pathways related to TNF-α, IL-6, and NFκB displayed the highest enrichment scores in transcriptome analysis (Fig. 2C,E). Another important result emerging from the human data is the activation of NK cells, probably facilitated by IFN-γ (Fig. 2C). Regarding lymphocytes in terms of IR injury, CD4^+^ T cells are usually considered most relevant^9^. Involvement of NK cells has also been reported^9^, albeit more rarely. Interestingly, we found transcription of genes related to NK cell activation being significantly more amplified in WT mice than in mice deficient of functioning MSTN when exposed to IR. This aligns well with the observed negative transcriptional regulation of IL-12 production in *Mstn*^*LnLn*^ mice due to IR. IL-12 physiologically activates NK cells and lymphocytes and stimulates the production of IFN-γ and TNF-α.

The main purpose of this work, however, was to investigate the protective capacity of MSTN deficiency in the setting of IR injury. First of all, experiments conducted with WT mice mirrored results observed after flap surgery in humans (Figs. 1, 3), rendering the used in vivo IR model highly suitable to examine the impact of the *Mstn* knockout. MSTN deficiency reduced neutrophil infiltration, myofiber deterioration, tissue edema, lipid peroxidation, and apoptosis (Fig. 3). In that regard it should be stated that the used antibody is directed against myostatin (GDF8) and GDF11. Due to their homology however, differentiation in immunofluorescence is not expedient. The hypothesized p38 MAPK implication was supported by absence of its up-regulation in *Mstn*^*Ln/Ln*^ mice after IR. Protective effects of MSTN inhibition have previously been reported in the heart. This was achieved using a soluble ACVR2B decoy receptor^19^ or by inhibiting ACTRII/TGFBR signaling^47^, respectively. Interestingly, the latter presented an underlying mechanism we identified ourselves during transcriptome analysis in mice: the mentioned up-regulation of *Atp2a2* in *Mstn*^*Ln/Ln*^ mice with concurrent down-regulation in WT mice, which was paralleled by up-regulation of SERCA2a (translational product of *Atp2a2*) in the setting of cardioprotection after myocardial infarction in mice^47^. Additionally, Zhu et al.^48^ had previously linked SERCA2a activity to p38 MAPK signaling: in 2017, they studied the effects of IR on rat cardiac function. It was found that during IR, p38 MAPK pathway activation eventually suppresses the activity of SERCA2a which reinforces calcium overload and mitochondrial membrane potential imbalance leading to cardiomyocyte apoptosis. This bridges the gap between myostatin inhibition, p38 MAPK signaling, and enhanced SERCA2a activity. Noteworthy, our findings paralled previous findings in cardiac muscle.

Scanning for differently regulated DEGs in WT and *Msnt*^*Ln/Ln*^ mice as a result of IR also identified further mediators (*Krt8, Krt18, Myh7b,* and *Npr3*) that may confer protective effects of MSTN deficiency. The first two ones code for keratin 8 and keratin 18, proteins that are usually found in intermediate filaments of epithelial cells. Interestingly, their ectopic expression in the heart has been linked to cardioprotective effects^49^. This is in line with our earlier observations in skeletal muscle, which showed up-regulation of *Krt8* and *Krt18* in *Msnt*^*Ln/Ln*^ mice but down-regulation in WT mice after IR. Additionally, keratin 8 is known to interact with p38 MAPK^50^. The protein product of *Myh7b* (miR-499) was also up-regulated in *Msnt*^*Ln/Ln*^ mice. In the heart, miR-499 could prevent cardiomyocyte apoptosis and reduced the infarct size of myocardial infarction in mice^51^. In murine skeletal muscle, miR-499 may drive myogenic differentiation of satellite cells and inhibit their adipogenic differentiation^52^ which also aligns well with the amplification of hallmark gene set “myogenesis” in *Msnt*^*Ln/Ln*^ mice after IR without any enrichment in WT mice (Fig. 4D). Another protein potentially capable of inhibiting apoptosis in cardiomyocytes is natriuretic peptide receptor 3 (NRP3). While up-regulated in *Msnt*^*Ln/Ln*^ mice and down-regulated in WT mice after IR in this work, previous in vitro studies showed enhanced levels of caspase 3, 8, and 9 due to knockdown of NPR3 in H9C2 cells, and protective properties of NPR3 regarding H_2_O_2_ induced cell death^53^.

In contrast to earlier findings, we could not observe any influence of MSTN deficiency on ROS generation. Considering the results of this work and presented signaling pathways—including p38 MAPK circuits—one could hypothesize that inhibition of MSTN mitigates ROS production^18^. And indeed, this connection has previously been reported^15^. Contrarily, a recent publication stated that myostatin knockout induced apoptosis in human cervical cancer cells which was mediated via ROS generation^54^. In the presented work, however, ROS related pathways did not differ significantly between WT and *Mstn*^*Ln/Ln*^ mice. We therefore speculate that the connection between MSTN and ROS generation is far more complex and highly context dependent. As another important finding, increased activity of genes related to myogenesis, angiogenesis and notch signaling in vivo arising from MSTN deficiency suggests enhanced regenerative capacity in addition to reduced susceptibility to IR injury. In other words, besides causing attenuated tissue deterioration and cell death, MSTN deficiency seems to result in up-regulation of mechanisms that restore tissue integrity after the damaging process has occurred. Up-regulation of *Mymx* in *Mstn*^*Ln/Ln*^ mice and down-regulation in WT mice after IR further supports this assumption. *Mymx* codes for myomerger, which, together with myomaker, is one of the two fundamental fusion proteins required for skeletal muscle development^55^. While myomaker is also essential for muscle regeneration^56^, the role of myomerger has not been studied in the same depth. Yet, observed regulation of myomerger supports the idea of enhanced tissue regeneration emerging from MSTN deficiency.

In conclusion, this study illuminated the occurrence of IR injury in reconstructive free flap surgery and highlighted potential beneficial effects of MSTN deficiency in these as well as similar scenarios. We further examined reasons linking absence of myostatin to reduced cell death and enhanced tissue regeneration. Thereby, we identified p38 MAPK, SERCA2a, keratin 8 and keratin 18, miR-499, NRP3, and myomerger; either individually or interdependently and collectively as potential targets regarding IR injury. We would like to encourage further studies to focus on these proteins, even if not in combination with MSTN. Additionally, we identified a potential role of NK cells in skeletal muscle IR injury that needs further investigation.

## Materials and methods

### Human specimen collection

**Figure 5:**
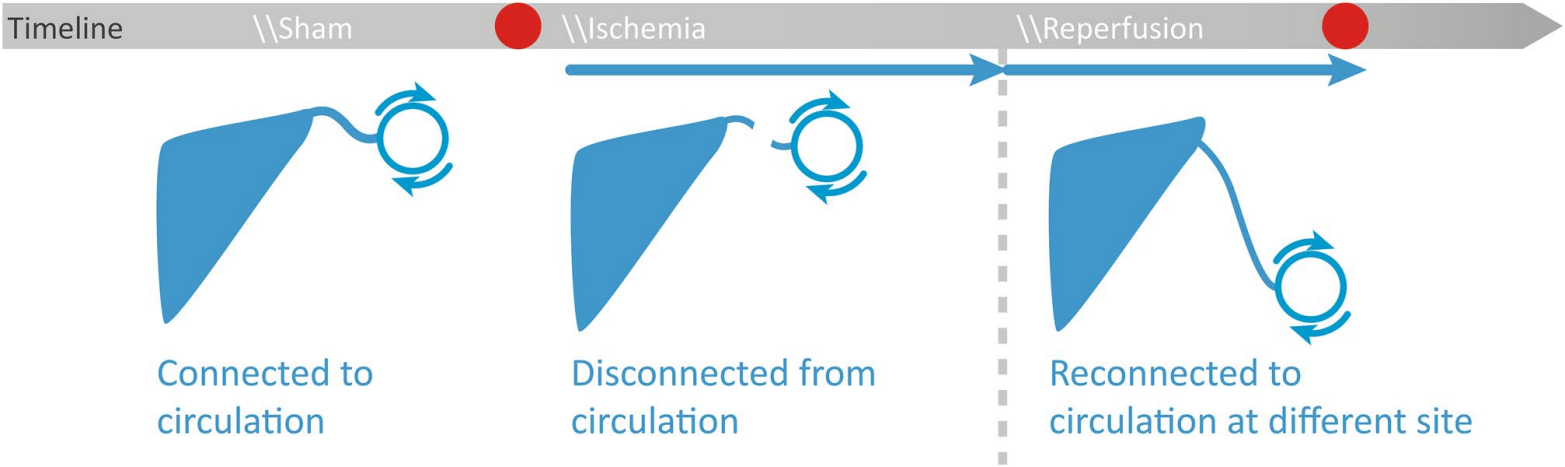
Timeline of specimen harvest during free flap surgery.

All procedures were performed in accordance with the ethical committee of the Ruhr University Bochum after approval (approval number: 16-5932-BR). Informed consent was obtained from all subjects participating in the study. Human skeletal muscle tissue was harvested during planned latissimus dorsi free flap surgery (Fig. 5). During each surgery two tissue specimens were taken: the first one immediately before blood circulation of the latissimus dorsi was detached (Control) and the second one shortly before the procedure was finished (Ischemia/Reperfusion). The specimens were immediately placed into liquid nitrogen for gene expression analysis or formaline (4%) for histology and immunofluorescence.

### Histology and immunofluorescence

Tissue samples were stained with haematoxylin and eosin for analysis of tissue damage and neutrophil infiltration. For immunofluorescence stainings, sections and chamber slides were incubated at 58 °C for 1 h and subsequently rehydrated and incubated with 0.125% Proteinase K for 15 min at 37 °C. After a short washing step with PBS, sections and cells on chamber slides were permeabilized with 0.1% Tween 20 for 4 min and treated with blocking solution for 1 h. Incubation with primary antibodies (Supplementary Dataset S11) in blocking solution followed overnight at 4 °C. After washing with PBS, a goat biotinylated secondary antibody was used for detection. For mouse monoclonal antibodies conjugated mouse IgGκ binding proteins were used instead of the secondary antibody. All sections have been counterstained with DAPI. Sections were subsequently mounted with Fluoromount Aqueous Mounting Medium (Sigma Aldrich). Images for immunofluorescence were taken with a fluorescence microscope (Olympus IX3-Series). By using the Adobe Magic Wand Tool (settings: tolerance 60%; noncontiguous) in Adobe Photoshop CS6 (https://www.adobe.com/products/photoshop.html), immunohistochemical positive stained pixels were selected automatically and divided by countable nuclei. Afterwards a mean value was calculated. A detailed list of antibodies used can be found in the supplemental material Supplementary Dataset S11). For measuring the infiltration of neutrophils, four regions of interest per section (stained with HE) were chosen (1000 × 1000 Px), and neutrophils were counted by three independent observers. For the morphometric analysis, an unbiased sampling procedure was applied. The fraction of normal muscle cells was calculated by measuring the cross-sectional diameters of fibers in four regions of interest in transverse sections (stained with HE) as reported before^57^. Cells with a diameter within 10% of the control group values were considered “normal cells”. Percentage of normal cells was calculated accordingly.

### RNA preparation

After harvesting, muscle specimens were immediately stored in cryovials and shock frozen in liquid nitrogen. Total RNA was then isolated using the RNeasy Fibrous Tissue Mini Kit (Qiagen, Hilden, Germany). RNA-quality was assessed via NanoDrop. A 260/280 ratio ≥ 2 and 260/230 ratio of 1.8–2.2 were considered sufficient for further analysis. An amount of 100 ng of every total RNA sample was hybridized to an individual Agilent whole genome expression microarray (Human GE 4 × 44K, v2 G4845A, AMADID 026652, Agilent Technologies) according to the Agilent single color protocol. RNA labeling, hybridization and washing were carried out according to the manufacturer’s instructions. Images of hybridized microarrays were acquired with a DNA microarray scanner (Agilent G2505B) and features were extracted using the Agilent Feature Extraction image analysis software (AFE) version A.10.7.3.1 with default protocols and settings. The AFE algorithm generates a single intensity measure for each feature, referred to as the total gene signal (TGS), which was used for further data analyses using the GeneSpring GX software package version 14.9.1. AFE-TGS were normalized by the quantile method. Subsequently, data were filtered on normalized expression values^17^. The gene expression data from our study have been deposited in the NCBI's Gene Expression Omnibus (GEO) database (accession number GSE118380).

### Breeding and genotyping of the C57BL/6J-Mstn^Ln/Ln^ strain

Heterozygote C57BL/6J-*Mstn*^*Ln*^*/*^*J*^ mice were obtained from Jackson Laboratory (Maine, USA, Stock No 009345). Multidose *N*-ethyl-*N*-nitrosourea (ENU) treatment was used to induce mutations (T → C transition in the second intron two base pairs downstream of exon 2 in the myostatin gene) in founder C57BL/6J mice which resulted in improper splicing and a loss-of-function allele called the lean (Ln) allele of *Mstn* (*Mstn*^*Ln*^). Mice were then bred to obtain homozygote C57BL/6J-*Mstn*^*Ln/Ln*^ offsprings. Homozygote mice were identified using a qPCR genotyping assay consisting of a forward primer (5′ ACA GCC TGA ATC CAA CTT AGG 3′), a reverse primer (5′ AAA TGT AAC ACG GTT GCT AGA ATG 3′), a probe for the wild type allele (5′-HEX AGA TGG GCT GGT AAG TGA TAA CT BHQ1-3′), and a probe for the lean allele (5′-FAM GAT GGG CTG GCA AGT GAT AAC BHQ1-3′).

### Animal surgery

All animal experiments were approved by the IACUC LANUV NRW (The Ministry for Environment, Agriculture, Conservation and Consumer Protection of the State of North Rhine-Westphalia), Permit Number: 84-02.04.2016.A045, and the study was carried out in accordance with the ARRIVE guidelines. C57BL/6J and C57BL/6J-Mstn^lean^ mice were obtained (The Jackson Laboratory, Maine, USA) and kept with unlimited access to water and standard laboratory chow at a 12 h light/dark cycle. Littermates of both sexes at the median age of 12 (± 1) weeks were used for all experiments. All interventional procedures were performed under inhalation anesthesia with isoflurane (3% for induction, 0.8–1.8% for maintenance) and buprenorphine (0.05 mg/kg s.c.). To maintain a body temperature of 37 °C mice were restrained on a heating pad for the whole duration of the intervention. C57BL/6J wild type mice (24–27 g) and C57BL/6J-*Mstn*^*Ln/Ln*^ mice (28–33 g) were randomized in an experimental (IR) and a control (C) group resulting in four groups: WT-C (1), WT-IR (2), *Mstn*^*Ln/Ln*^-C (3), and *Mstn*^*Ln/Ln*^-IR (4). In the IR groups, ischemia was induced as previously described^28^. Hindlimb perfusion was interrupted for 2 h, followed by 4 h of reperfusion. Subsequently, mice were euthanized in accordance with the American Veterinary Medical Association Guidelines for the Euthanasia of Animals, and the tissue was harvested. Control group mice were anesthetized for 2 h without any interventions and euthanized 4 h later.

### Edema weight and wet to dry ratio

Immediately after euthanasia the leg was cut off in toto, weighed and placed in an oven at 55 °C for 48 h. Subsequently, the leg was weighed again. From here, two calculation methods were used. For the first one, the difference in edema weight increase (in mg) within one group (C57BL/6J or C57BL/6J-*Mstn*^*Ln/Ln*^) before and after drying was compared with the other group (edema weight). For the second method, the weight within each group after drying was divided by the weight before drying, resulting in values with the unit “percent” (wet to dry ratio and wet to dry ratio increase). Then the difference in wet to dry ratio increase between both groups was calculated. This was done to account for the heavier weight of C57BL/6J-*Mstn*^*Ln/Ln*^ in comparison to C57BL/6J mice.

### Apoptosis array

For cell lysate preparation, equal amounts of 3 samples per group were suspended in Lysis Buffer 17 provided with the Proteome Profiler Mouse Apoptosis Array (R&D systems, Minneapolis, Minnesota, USA). After homogenization with the T 10 basic ULTRA-TURRAX (IKA, Staufen im Breisgau, Germany) and centrifugation at 12,000 rpm, supernatant was transferred to a fresh tube, and protein concentration was determined by using the BCA Assay (ThermoFisher Scientific, Waltham, USA). Cell lysates (400 µg) were added to the array membranes and subsequent steps were carried out according to the manufacturer’s instructions. Membranes were then exposed to X-ray film for 2 min each. For data analysis, X-ray films were scanned, and pixel density was quantified in Adobe Photoshop after subtracting an average background signal from each spot.

### Statistics

After testing for homo- or heteroscedasticity (F Test), p values for pairwise comparisons were analyzed via two-tailed unpaired t test with (heteroscedasticity) or without (homoscedasticity) Welch’s correction. Multi-group comparisons were carried out using ANOVA followed by Tukey’s post-hoc test (homoscedasticity) or Brown-Forsythe and Welch ANOVA followed by Dunnett T3 post-hoc test (heteroscedasticity). All analyses were performed using GraphPad PRISM (version: 8.3.0; Graphpad Software, Inc, California, USA). Statistical significances were set at a p value < 0.05. For identification of DEGs, only entities where at least 2 out of the total number of samples had values within the selected cut-off (50th–100th percentile) were further included in the data analysis process. Using the GeneSpring GX software package version 14.5, pairwise comparisons of filtered and normalized single color array data were used to identify differentially expressed genes (moderated t test). The p values were adjusted for multiple testing according to Benjamini and Hochberg (false detection rate—FDR) and results were considered statistically significant at adjusted p values below 0.05. Lastly, only mRNAs with a fold change ≥ 1.7 in the microarray analyses were further considered. For Gene Set Enrichment Analysis, FDR significance thresholds are provided within the figure legends. For figure preparation Adobe Illustrator version 24.1.1 (https://www.adobe.com/products/illustrator.html) was used.

### Study approval

The authors certify that they comply with the ethical guidelines for authorship and publishing. The study was performed after approval from the ethical committee of the Ruhr University Bochum (approval number: 16-5932-BR) and in accordance with the Declaratation of Helsinki principles. Written informed consent was obtained from every participant, and participants were identified by numbers. All animal experiments were approved by the IACUC LANUV NRW (The Ministry for Environment, Agriculture, Conservation and Consumer Protection of the State of North Rhine-Westphalia), Permit Number: 84-02.04.2016.A045.

## Supplementary Information

Supplementary Information 1.

Supplementary Information 2.
