# Supplementary material for: Deficiency of myostatin protects skeletal muscle cells from ischemia reperfusion injury
Source: Sci Rep. 2021 Jun 15;11:12572. doi: 10.1038/s41598-021-92159-2 (PMC8206371; doi:10.1038/s41598-021-92159-2)
Supplement: Supplementary file 1 — Supplementary Information 1. [file 41598_2021_92159_MOESM1_ESM.pdf]

Supplementary Information for "Deficiency of myostatin protects skeletal muscle cells from ischemia reperfusion injury"

Christoph Wallner,[1*] Marius Drysch,[1*] Mustafa Becerikli,[1] Sonja Verena Schmidt,[1] Stephan Hahn,[2] Johannes Maximilian Wagner,[1] Felix Reinkemeier,[1] Mehran Dadras,[1] Alexander Sogorski,[1] Maxi von Glinski,[1] Marcus Lehnhardt,[1] Björn Behr[1]

[1] Department of Plastic Surgery, BG University Hospital Bergmannsheil, Ruhr University Bochum, Bürkle-de-la-Camp Platz 1, 44789 Bochum, Germany

[2] Department of Molecular Gastrointestinal Oncology, Ruhr University Bochum, Universitätsstraße 150, 44780 Bochum, Germany

*Both authors share the first author position

Corresponding author: Björn Behr, MD

Email: bjorn.behr@rub.de

This PDF file includes:

Supplementary Figures S1 to S5

Other supplementary materials for this manuscript include the following:

Supplementary Datasets S1 to S11

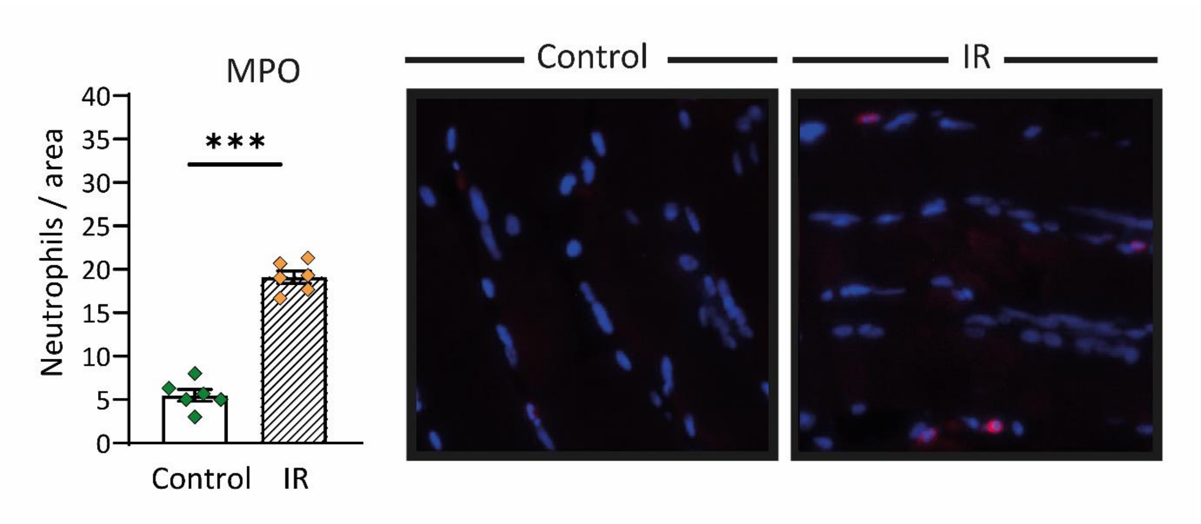

Fig. S1: Neutrophils per area measured by staining of myeloperoxidase (MPO). Right panel shows representative images of immunofluorescence analysis before and after IR in human specimens harvested during free flap surgery. P-values were calculated by ANOVA followed by multiple test comparison via Tukey's post-hoc test (*** < 0.001).

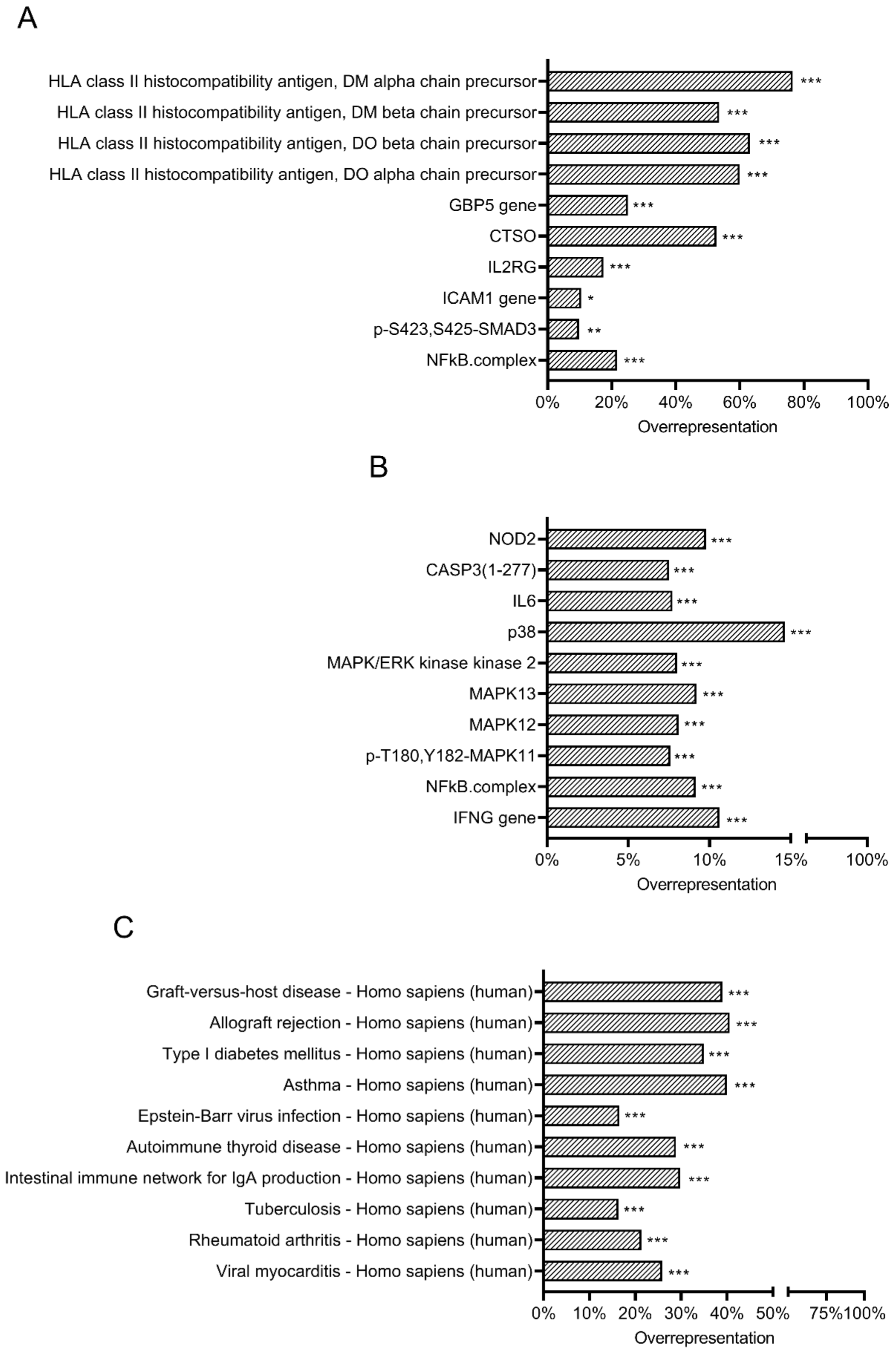

Fig. S2: Transcriptional analysis of human skeletal muscle IR. ConsensusPathDB overrepresentation analysis of DEGs in human skeletal muscle specimen harvested during free flap surgery based on **(A)** Network neighbourhood-based entity sets (NESTs) with radius 1, NESTs with radius 2 **(B)**, and Kyoto Encyclopedia of Genes and Genomes (KEGG) pathway database **(C)**. P-values were calculated using a hypergeometric distribution; * < 0.05, ** < 0.01, *** < 0.001.

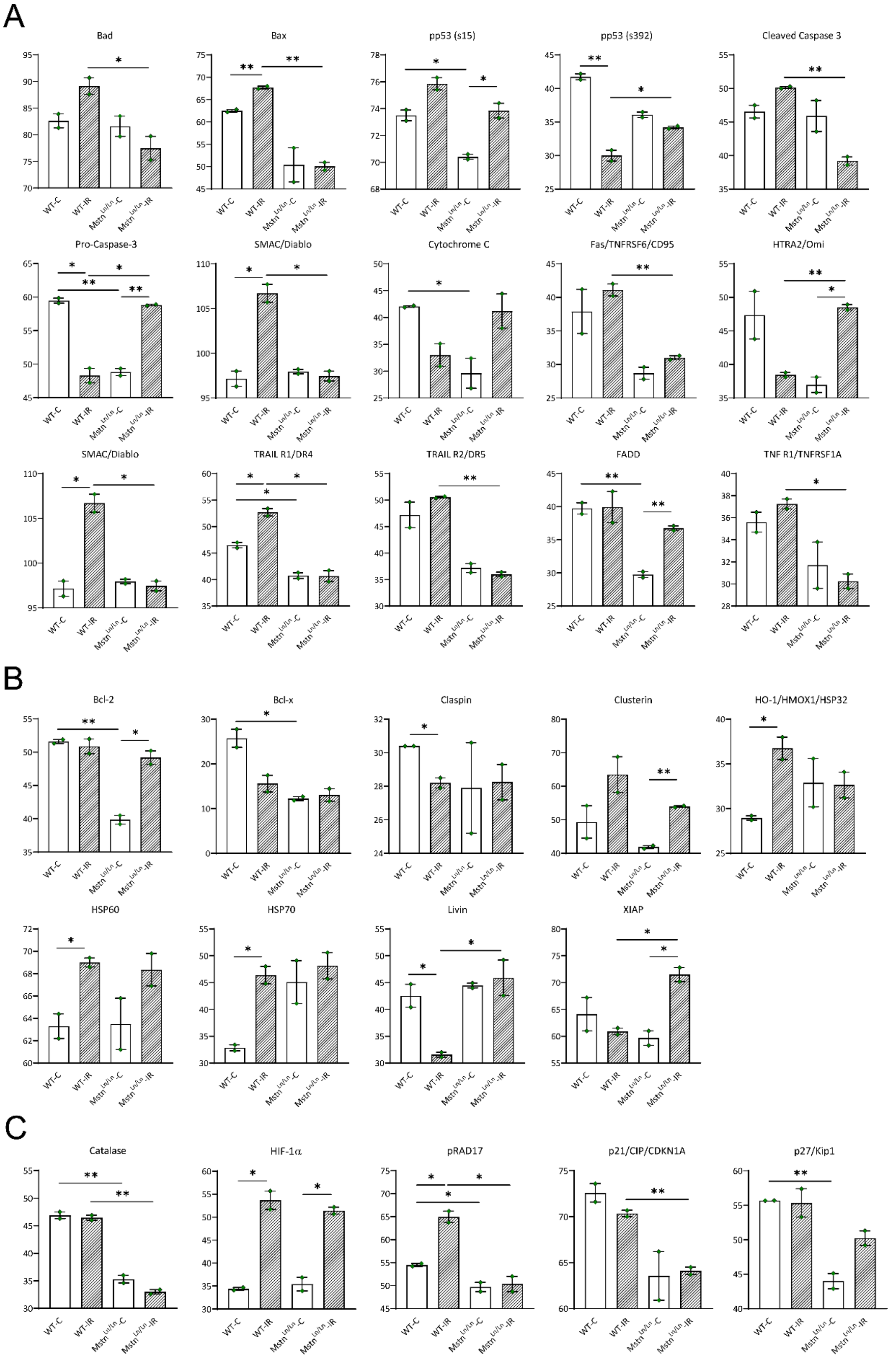

Fig. S3: Quantification of apoptotic activity in skeletal muscle specimens from C57BL/6J and C57BL/6J-*Mstn*$^{Ln/Ln}$ mice before and after IR. (A) Proteins mainly associated with pro-apoptotic **(A)** or anti-apoptotic **(B)** activity. **(C)** Proteins related to cell cycle regulation and oxygenation. P-values were calculated by ANOVA followed by multiple test comparison via Tukey's post-hoc test; * < 0.05, ** < 0.01, *** < 0.001.

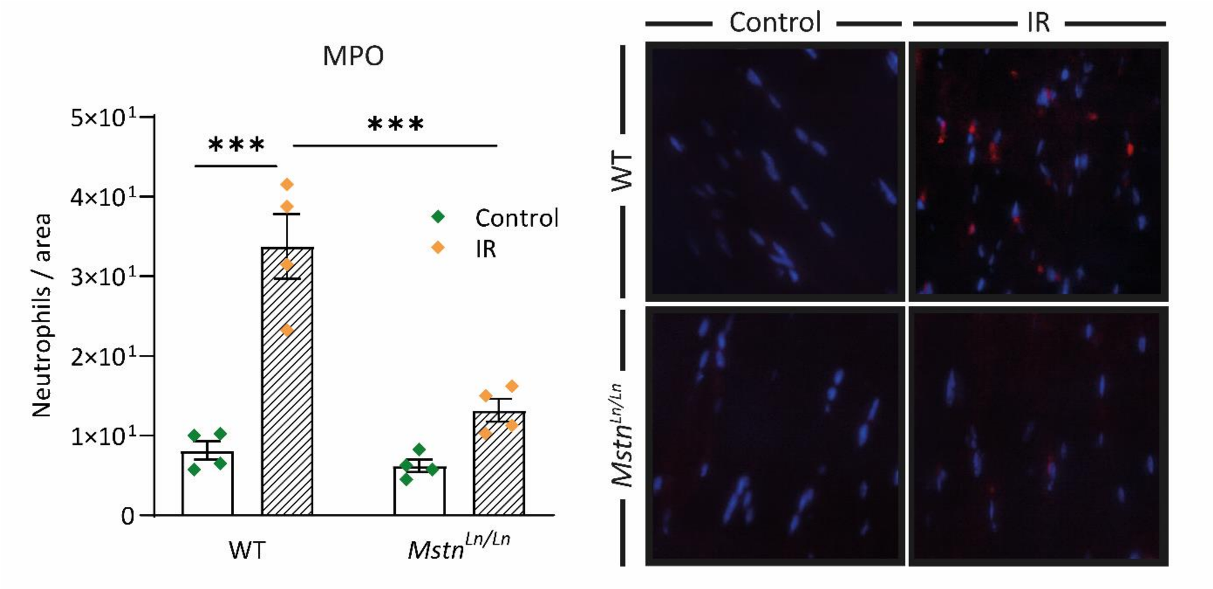

Fig. S4: Neutrophils per area measured by staining of myeloperoxidase (MPO). Right panel shows representative images of immunofluorescence analysis before and after IR in C57BL/6J and C57BL/6J-*Mstn*$^{Ln/Ln}$ mice, respectively. P-values were calculated by ANOVA followed by multiple test comparison via Tukey's post-hoc test (*** < 0.001).

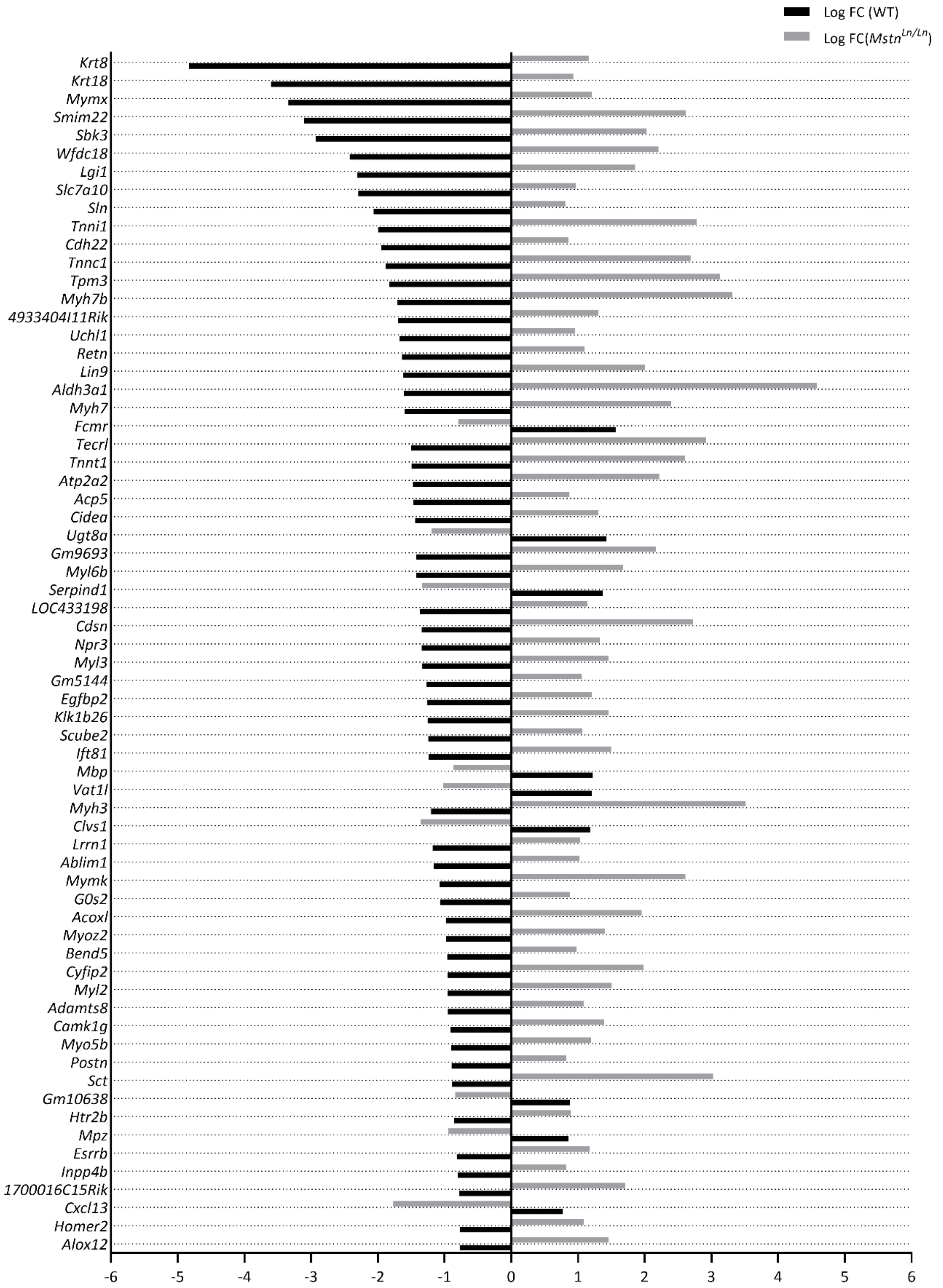

Fig. S5: Potential target proteins mediating GDF8 deficiency related IR protection in murine skeletal muscle. DEGs that are regulated differently when comparing transcriptional activity before and after IR in C57BL/6J and C57BL/6J-*Mstn*$^{Ln/Ln}$ mice, respectively. X-Axis depicts log fold-change. FDR < 0.05 for all depicted genes (Benjamini-Hochberg).
